# Data from a proteomic baseline study of Assemblage A in *Giardia duodenalis*

**DOI:** 10.1016/j.dib.2015.08.003

**Published:** 2015-08-19

**Authors:** Samantha J. Emery, Ernest Lacey, Paul A. Haynes

**Affiliations:** a Department of Chemistry and Biomolecular Sciences, Macquarie University, North Ryde, NSW 2109, Australia; b Microbial Screening Technologies Pty Ltd, Smithfield, NSW 2165, Australia

**Keywords:** Assemblage A, *Giardia duodenalis*, Label-free quantitative shotgun proteomics, Variant Surface Protein, Variable genome, Parasite proteomics

## Abstract

Eight Assemblage A strains from the protozoan parasite *Giardia duodenalis* were analysed using label-free quantitative shotgun proteomics, to evaluate inter- and intra-assemblage variation and complement available genetic and transcriptomic data. Isolates were grown in biological triplicate in axenic culture, and protein extracts were subjected to in-solution digest and online fractionation using Gas Phase Fractionation (GPF). Recent reclassification of genome databases for subassemblages was evaluated for database-dependent loss of information, and proteome composition of different isolates was analysed for biologically relevant assemblage-independent variation. The data from this study are related to the research article “*Quantitative proteomics analysis of Giardia duodenalis Assemblage A – a baseline for host, assemblage and isolate variation*” published in Proteomics (Emery et al., 2015 [1]).

**Specifications table**

**Table:** 

Subject area	Biology
More specific subject area	Quantitative proteomic data of 8 *Giardia duodenalis* Assemblage A isolates using gas phase fractionation and normalised spectral abundance factors (NSAF).
Type of data	Table, Figure, Supplementary Tables
How data was acquired	Protein extracts from biological triplicates were digested in solution, and fractionated online using GPF with mass range fraction optimised for the *G. duodenalis* A1 subassemblage genome. Data was acquired on a LTQ-XL Linear Ion Trap (Thermo).
Data format	Raw data, reproducibly identified proteins.
Experimental factors	8 *G. duodenalis* strains grown in Axenic culture from animal and human hosts, covering both subassemblage A1 and A2 to analyse isolate variation. Data was searched against both A1 subassemblage genome database and recently released A2 subassemblage database to compare database-specific losses.
Experimental features	Sample triplicates were combined to produce reproducibly identified proteins and spectral counts of each protein were used to calculate NSAF values for each protein.
Data source location	Sydney, NSW, Australia
Data accessibility	Data is available from http://www.ebi.ac.uk/pride/archive/projects/PXD001272 and will also be made available through the giardiadb.org website later in 2015.

**Table:** 

*Value of the data*
• First proteomic baseline for taxonomy and isolate variation in Assemblage A strains. • Provides proteome coverage of isolates from animal and human hosts, both A1 and A2 subassemblages, with an emphasis on Australian isolates. • Evaluates database-dependent losses based on new genome reclassifications and releases in Assemblage A. • Identifies sources of inter- and intra-assemblage A isolate variation and its impacts.

## 1 Experimental design, materials and methods

### 1.1 Isolate selection, axenic culture, protein extraction and digestion

Eight Assemblage A strains [1], including the A1 genome strain, were assembled from animal and human infections, previously characterised in the literature according to karotype [2,3], subassemblage [4], virulence [2], geographic variation [5,6] and drug resistance [7]. The full description of strains can be seen in Table 1.

*G. duodenalis* strains were cultured in triplicate axenically in TYI-S33 media supplemented with 10% newborn calf serum and 1% bile as previously described [8] and harvested from confluent cultures in late log-phase. Trophozoites were harvested by centrifugation, washed twice in ice-cold PBS to remove media traces [9] and pellets of 10^8^ trophozoites were extracted into 1 mL ice-cold SDS sample buffer containing 1 mM EDTA and 5% beta-mercaptoethanol, then disulphides were reduced at 75 °C for 10 min. Trophozoite protein extracts were centrifuged at 0 °C at 13,000×*g* for 10 min to remove debris, and protein concentration was measured by BCA assay (Pierce). A 500 µg protein pellet was extracted using methanol–chloroform precipitation [10] and in-solution digestion was performed using a modified filter aided sample preparation (FASP) [11]. After peptide extraction all samples were dried using a vacuum centrifuge and reconstituted to 60 µL with 2% formic acid, 2% 2,2,2-trifluorethanol (TFE).

### 1.2 Nanoflow LC-MS/MS using gas phase fractionation

Optimised gas phase fractionation (GPF) mass ranges were calculated using the 2.5 release of the *G. duodenalis* WB genome for Assemblage A from giardiaDB.org [12]. Charge states +2 and +3 were considered as well as carbamidomethyl as a cysteine modification, and 4 mass ranges were calculated over 400–2000 amu. The mass ranges were as following: the low mass range was 400–518 amu, the low-medium mass range was 518–691 amu, the medium-high mass range was 691–988 amu and the high mass range was 988–2000 amu. Each FASP protein digest for the triplicates of each strain were analysed by nanoLC-MS/MS on an LTQ-XL linear ion trap mass spectrometer (Thermo, San Jose, CA). Peptides were separated on a 150×0.2 mm I.D fused-silica column packed with Magic C18AQ (200 Å, 5 µm diameter, Michrom Bioresources, California) connected to an Advance CaptiveSpray Source (Michrom Bioresources, California). Each FASP protein digest was analysed as 4 repeat injections, with the mass spectrometer scanning for 180 min runs for each of the four calculated mass ranges. Samples were injected onto the column using a Surveyor autosampler, followed by an initial wash step with buffer A (0.1% v/v formic acid, 1 mM ammonium formate, 0.2% v/v methanol) for 4 min followed by 150 µL/min for 2 min. Peptides were eluted from the column with 0–80% buffer B (100% v/v ACN, 0.1% v/v formic acid) at 150 µL/min for 167 min finished by a wash step with buffer A for 6 min at 150 µL/min. Spectra in the positive ion mode were scanned over the respective GPF ranges and, using Xcalibur software (Version 2.06, Thermo), automated peak recognition, dynamic exclusion and MS/MS of the top six most-intense ions at 35% normalisation collision energy were performed.

### 1.3 Database searching for protein/peptide information

The LTQ-XL raw output files were converted into mzXML files and searched against the Giardiadb.org 4.0 release of *G. duodenalis* strain Assemblage A1 and A2 genome using the global proteome machine (GPM) software (version 2.1.1) and the X!Tandem algorithm. The 4 fractions for the GPF of each replicate were processed sequentially with output files generated for each individual fraction, and a merged, non-redundant output file for protein identifications with log(*e*) values<−1. Peptide identification was determined using MS and MS/MS tolerances of +2 Da and +0.4 Da. Carbamidomethyl was considered a complete modification, and partial modifications considered included oxidation of methionine and tryptophan.

### 1.4 Data processing and quantitation

**Fig. 1:**
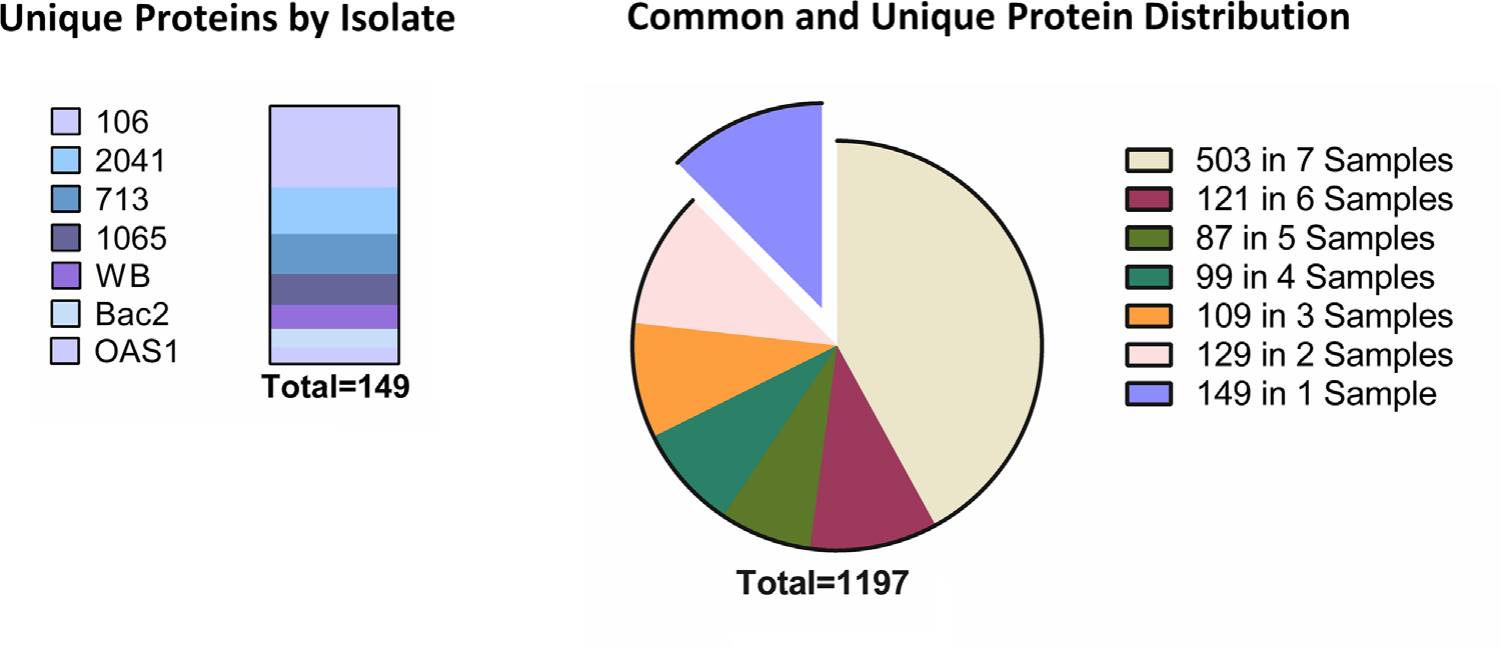
Distribution of shared and unique proteins in the A1 subassemblage between the 1197 non-redundant proteins identified within the seven isolates analysed. The 1197 proteins were reproducibly identified in at least one isolate, with 149 (12.4%) of these proteins identified within only one isolate, and therefore considered to be uniquely expressed. Part A (left) shows the distribution of these 149 uniquely expressed proteins by isolate in the seven A1 isolates analysed in this study. Part B (right) shows the distribution of the shared proteins between the seven subassemblage A1 isolates. A total of 503 (42%) proteins were identified in all seven isolates examined in this study, and are considered common between isolates of the A1 subassemblage. The remaining segments indicates proteins common within decreasing numbers of isolates, while the final elevated segment indicates the 149 isolate-unique proteins.

The output from the GPM software (version 2.1.1) [13,14] constituted low stringency protein and peptide identifications, and was used to assess experimental consistency. These data were further processed using the Scrappy software package [15], which combines biological triplicates into a single list of reproducibly identified proteins, which we define in this study as those proteins present reproducibly in all three replicates of at least one strain, with a total spectral count (SpC) of ≥5 [15]. Reversed database searching was used for calculating peptide and protein false discovery rates (FDRs) as previously described [15]. Complete protein and peptide data for replicates, including database-dependent losses are shown in Supplementary data 1, Table 1 and in *Giardia* specific gene-families in Supplementary data, Table 2. Protein abundance was calculated using NSAF values [16]. Distribution of reproducibly identified proteins by strain can be viewed in Fig. 1. The mass spectrometry proteomics data have been deposited to the ProteomeXchange Consortium [17] via the PRIDE partner repository with the dataset identifier PXD001272.

## 2 Direct link to deposited data

Data is available through the PRIDE proteomics database through the following link http://www.ebi.ac.uk/pride/archive/projects/PXD001272 and will also be made available through the giardiadb.org website later in 2015.

## 3 Conflict of interest

The authors declare that there is no conflict of interest on any work published in this paper.

## Figures and Tables

**Table 1:** Classification information for the eight *G. duodenalis* strains used in this study including subassemblage, geographic origin, and the host species the strain was isolated from. Strain identification coincides with those previously published in the literature.

**Strain**	**Assemblage**	**Origin**	**Host source**
BRIS/83/HEPU 106	A1	Brisbane, Australia	Human
BRIS87/HEPU/713	A1	Brisbane, Australia	Human
OAS1	A1	Canada	Sheep (*Ovis aries*)
Bac2	A1	Australia	Cat (*Felis catus*)
BRIS/95/HEPU/2041	A1	Victoria, Australia	Cockatoo (*Cacatua galerita*)
BRIS/89/HEPU/1065	A1	Brisbane, Australia	Human
WB*	A1	Afghanistan	Human
BRIS/89/HEPU/1003	A2	Brisbane, Australia	Human

⁎ Assemblage A1 genome strain (ATCC 50803).

## Footnotes

Appendix A Supplementary data associated with this article can be found in the online version at doi:10.1016/j.dib.2015.08.003.
