# Supplementary material for: Data from a proteomic baseline study of Assemblage A in Giardia duodenalis
Source: Data Brief. 2015 Aug 19;5:23–7. doi: 10.1016/j.dib.2015.08.003 (PMC4556777; doi:10.1016/j.dib.2015.08.003)
Supplement: Supplementary file 1 — Supplementary data [file mmc1.docx]

**A) A1 Genome**

| **Sample & Subassemblage** |  | **Low Stringency Peptide Count** | | | **Average No. Peptide (±%RSD)** | **No. R.I.⃰ Proteins Common to 3 Replicates** | **R.I.⃰ Protein FDR (%)** | **R.I.⃰ Peptide FDR(%)** |
| --- | --- | --- | --- | --- | --- | --- | --- | --- |
|  |  | **Replicate 1** | **Replicate 2** | **Replicate 3** |  |  |  |  |
| **BRIS/83/HEPU 106** | A1 | 23016 | 21198 | 22837 | 22350 ± 4.48% | 895 | 0.45 | 0.08 |
| **BRIS87/HEPU/713** | A1 | 20558 | 19945 | 18893 | 19799 ± 4.25% | 798 | 0.50 | 0.12 |
| **OAS1** | A1 | 21111 | 21209 | 18288 | 20203 ± 8.21% | 716 | 0.42 | 0.06 |
| **Bac2** | A1 | 20097 | 19285 | 19629 | 19670 ± 1.99% | 701 | 0.29 | 0.06 |
| **BRIS/95/HEPU/2041** | A1 | 21724 | 20807 | 21635 | 21389 ± 2.36% | 836 | 0.72 | 0.15 |
| **BRIS/89/HEPU/1065** | A1 | 18635 | 20740 | 20547 | 19974 ± 5.83% | 728 | 0.27 | 0.04 |
| **WB** | A1 | 21227 | 20699 | 21003 | 20976 ± 1.26% | 769 | 0.39 | 0.90 |
| **BRIS/89/HEPU/1003** | A2 | 18296 | 20103 | 20179 | 19526 ± 5.46% | 668 | 0.30 | 0.07 |

**B) A2 Genome**

| **Sample & Subassemblage** |  | **Low Stringency Peptide Count** | | | **Average No. Peptide (±%RSD)** | **No. R.I.⃰ Proteins Common to 3 Replicates** | **R.I.⃰ Protein FDR (%)** | **R.I.⃰ Peptide FDR(%)** |
| --- | --- | --- | --- | --- | --- | --- | --- | --- |
|  |  | **Replicate 1** | **Replicate 2** | **Replicate 3** |  |  |  |  |
| **BRIS/83/HEPU 106** | A1 | 21772 | 21111 | 23043 | 21975 ± 4.47% | 864 | 0.81 | 0.17 |
| **BRIS87/HEPU/713** | A1 | 19487 | 18916 | 18168 | 18857 ± 3.51% | 775 | 0.90 | 0.19 |
| **OAS1** | A1 | 19135 | 19476 | 17052 | 18554 ± 7.07% | 681 | 0.29 | 0.05 |
| **Bac2** | A1 | 18906 | 18127 | 18278 | 18437 ± 2.24% | 658 | 0.61 | 0.14 |
| **BRIS/95/HEPU/2041** | A1 | 20364 | 19511 | 20208 | 20028 ± 2.27% | 795 | 0.50 | 0.12 |
| **BRIS/89/HEPU/1065** | A1 | 18708 | 20812 | 20647 | 20055 ± 5.83% | 677 | 0.74 | 0.13 |
| **WB** | A1 | 20227 | 19844 | 19804 | 19958 ± 1.17% | 724 | 0.39 | 0.09 |
| **BRIS/89/HEPU/1003** | A2 | 19126 | 21036 | 21189 | 20450± 5.62% | 713 | 0.42 | 0.08 |

**R.I. stands for ‘Reproducibly Identified’.**

**Supplementary Table 1: Complete summary of peptide and protein identification data of *G. duodenalis* proteins across the eight strains analysed for both the subassemblage A1 and A2 reference genome. For the two tables A) shows the protein and peptide summary when searched against the subassemblage A1 genome sequence while B) shows the protein and peptide summary against the subassemblage A2 genome sequence.**

| **Strain** | **VSP** | **HCMP** | | | **Nek Kinase** | **Protein 21.1** | **TOTAL:** |
| --- | --- | --- | --- | --- | --- | --- | --- |
| BRIS/83/HEPU 106 | 13 | 4 | | 39 | | 53 | 109 (12.2%) |
| BRIS87/HEPU/713 | 1 | 2 | | 35 | | 44 | 82 (10.3%) |
| OAS1 | 7 | 5 | | 29 | | 39 | 80 (11.2%) |
| Bac2 | 23 | 4 | | 28 | | 36 | 91 (13.0%) |
| BRIS/95/HEPU/2041 | 33 | 2 | | 38 | | 52 | 125 (15.0%) |
| BRIS/89/HEPU/1065 | 37 | 7 | | 33 | | 40 | 117 (16.1%) |
| WB | 9 | 7 | | 31 | | 47 | 94 (12.2%) |
| BRIS/89/HEPU/1003* | 15 | 7 | | 27 | | 40 | 89 (13.3%) |
|  | |  | | |  |  | |
| **Genome Total:** | 186 | 59 | 179 | | | 243 |  |

**A) A1 Genome**

**B) A2 Genome**

| **Strain** | **VSP** | **HCMP** | | | **Nek Kinase** | **Protein 21.1** | **TOTAL:** |
| --- | --- | --- | --- | --- | --- | --- | --- |
| BRIS/83/HEPU 106 | 15 | 0 | | 11 | | 65 | 91 (10.5%) |
| BRIS87/HEPU/713 | 2 | 0 | | 8 | | 53 | 63 (8.1%) |
| OAS1 | 7 | 1 | | 7 | | 47 | 62 (9.1%) |
| Bac2 | 13 | 0 | | 7 | | 38 | 58 (8.8%) |
| BRIS/95/HEPU/2041 | 25 | 0 | | 2 | | 63 | 90 (11.3%) |
| BRIS/89/HEPU/1065 | 29 | 1 | | 7 | | 40 | 77 (11.4%) |
| WB | 11 | 0 | | 8 | | 48 | 67 (9.3%) |
| BRIS/89/HEPU/1003* | 38 | 0 | | 5 | | 52 | 95 (13.3%) |
|  | |  | | |  |  | |
| **Genome Total:** | 121 | 2 | 32 | | | 340 |  |

**Supplementary Table 2: Numbers of reproducibly identified proteins by major gene family in each strain from the *G. duodenalis* variable genome. Final column shows the total, both by number and as a percentage of all reproducibly identified proteins for the strain. For the two tables A) shows the numbers of reproducibly identified proteins by family when searched against the subassemblage A1 genome sequence while B) shows the numbers of reproducibly identified proteins by family when searched against against the subassemblage A2 genome sequence. The A2 strain BRIS/89/HEPU/1003 is distinguished with a ‘*’ next to its identifier**
